# Supplementary material for: Learning curve and surgical outcome of robotic assisted colorectal surgery with ERAS program
Source: Sci Rep. 2022 Nov 29;12:20566. doi: 10.1038/s41598-022-24665-w (PMC9709162; doi:10.1038/s41598-022-24665-w)
Supplement: Supplementary file 2 — Supplementary Information 2. [file 41598_2022_24665_MOESM2_ESM.zip › RAL Raw data-Ñ[▒K/138/HS_ñw╝╨░O▒Kñσ.pdf]

正式摘要(電子簽章：000000233508987)		
W83 / W83 ☐ 英文標題(注意:無英文姓名-請補病人基本檔!)	☐【讀取】憑證	

臺中榮民總醫院

出院病歷摘要

轉入號 : 轉出號 :

[redacted]

(8)流水編號	
(9)入院日期	110/12/29 (2021) 直腸外科 W83 - 102
(10)轉科日期	
(11)出院日期	111/01/03 (2022) 住院天數計 5 日

診斷

- 入院 :1. Rectum cancer
  2. Normal coronary artery tree by 2021/12/15 CAG
  3. HTN, under medication control
  4. Headache, follow up at KuanTien Neuro OPD
  5. DM, under medication control
  6. CKD
  7. L't 2nd finger s/p amputation over 30 years ago
- 出院 :1. Rectum cancer s/p Robotic assist LAR + loop ileostomy on 2021/12/30
  2. Normal coronary artery tree by 2021/12/15 CAG
  3. HTN, under medication control
  4. Headache, follow up at KuanTien Neuro OPD
  5. DM, under medication control
  6. CKD
  7. L't 2nd finger s/p amputation over 30 years ago

主訴

- Diarrhea over 6 months

病史

- This is a 76 years old male patient with history of
  1. Normal coronary artery tree by 2021/12/15 CAG
  2. HTN, under medication control
  3. Headache, follow up at KuanTien Neuro OPD
  4. DM, under medication control
  5. CKD
  6. L't 2nd finger s/p amputation over 30 years ago
  According to the patient, he suffered from diarrhea for about 6 months. He received colonoscopy with biospy at KuanTien in 2021/11, revealed Rectum adenocarcinoma. CT was arranged showed no obvious distnat metastasis. 2D echo showed left ventricular diastolic dysfunction. He came to our CRS OPD for second opinion, and admitted to CV for pre-OP CAG survey, showed Normal coronary artery tree. As mentioned above, this time he was admitted to our ward for scheduled operation.

體檢發現

- 124/68-, hr 80

  ECG: RBBB

  ECHO:
  LA (4.0 CM) & RV (3.1 CM) CHAMBER DILATATION
  CONC. LVH (1.1, 1.1 CM)
  AORTIC ROOT DILATATION (3.8 CM)
  AORTIC VALVE THICKENED WITH MILD AR
  MILD MR WITH REVERSE MV E/A RATIO
  MODERATE TR WITH PEAK/MEAN SYSTOLIC PG -- 37/26 MMHG
  SUGGEST PULMONARY HYPERTENSION
  DILATED PA TRUNK(-- 2.8 CM) WITH MILD PR

- THE LV EJECTION FRACTION IS 50 %
  LV CHAMBER SIZE (4.1, 2.3 CM)
  THE LV OF PROXIMAL 1/2 ANTEROLATERAL WALL & PROXIMAL 1/2 INFEROLATERAL WALL SHOWED MILD HYPOKINETIC MOTION
  MV E/E' SEPTAL– 9.5
  MV E/E' LATERAL – 6.2
  END
  - General appearance :
    - easy looking , conscious clear , without cardiopulmonary distress
    - Vital signs自動帶入date 體溫(BT) 脈搏(P) 呼吸(R) 血壓(BP) 身高(BH) 體重(BW) BMI
      20211229 36.5℃ 92次/分 18次/分 135/72mmHg 161.9cm 66.8kg 25.5
    - Vital signs: B.P. 135/72 mmHg , P.R. 92 /min , R.R. 18 /min , B.T. 36.5 ℃
  - HEENT :
    - Eyes: no anemic conjunctiva, no icteric sclera, normal EOM
    - ENT: no otorrhea, no rhinorrhea, no erythematous mucosa, no injected tonsil, no deformity
  - Neck: supple, freely movable, trachea midline,no deformity, no engorged jugular vein , no palpable thyroid or lymph node enlargement
  - Chest:
    - Inspection: Grossly normal, symmetric and fully expansion, no accessory muscle used, no operation scar, no spider angiomata, no gynecomastia
    - Palpation: no palpable mass or nodule, no subcutaneous emphysema, no tenderness
    - Ausculation: clear breathing sound
  - Heart:
    - Palpation: no thrill, no heave
    - Percussion: normal heart size
    - Ausculation: regular heart beat, normal S1, S2, no gallop, no murmur
  - Extremities: No deformity, full ranged of motion without limitation, no clubbing finger or nail cyanosis, no pitting edema, no ulcer or pigmentation, no obvious skin lesion, no varicose vein, no bone, joint or muscle tenderness

CRS CONDITION

- Karnofsky performance status: (100) Normal, no complaints, no evidence of disease
- PE of Abdomen:
- CEA: 11.32 ng/ml
- Imaging Studies:
- Colonoscope report: .
- Figures:
  1. colon & rectum

手術日期及方法

- 手術日期及方法20211230 1. Robotic (XI) assisted low anterior resection
  2. Intraoperative colonoscopy : (-).
  3. High ligation (+) : At IMA root (Via robotic clips). IMV was ligated via endoclip.
  4. Take down from the splenic flexure : (-)
  5. Robotic EndoGIA (45-4.3) X2 + EEA (33mm) + OpenGIA (80-3.8) X1 were applied to complete the side-to-end anastomosis
  6. Proximal cut end: > 10 cm, distal cut end: 4 cm
  7. No drainage tube was inserted
  8. ADHESION BARRIER FILM was placed
  9. Loop ileostomy (About 15~20cm from IC valve) was done over RLQ of abdomen

住院治療經過

- After admission, he received Robotic assist LAR+loop ileostomy on 2021/12/30. He start try bland I diet on 1/01 and try blnad II diet on 1/02. Foley catheter was removed on 1/02. Under relative stable condition, he discharged on 1/03, arrange CRS and CV OPD f/u.

合併症

一般檢查

- 累積報告 :

DATE	TIME	ALB	TP	BIL.T	BIL.D	ALKP	AST	ALT	LDH	AMY	LIPASE	NH3	Ferritin	UA

-

1101229	1907	4.3	6.7	0.50	0.12	58	15	18						6.7
1101231	0007	3.7		0.65		51		15						

DATE	TIME	WBC	RBC	HGB	HCT	MCV	PLT	BLST	NEUT	BAND	SEG	LYM	MON	EOS	BAS
1101229	1907	4050	4.18	13.2	40.1	95.9	205		56.7			31.4	7.9	3.5	0.5
1101231	0006	10050		12.1	37.3		197		87.7			9.2	3.0	0.0	0.1

DATE	TIME	NA	K	CL	BUN	CREAT	eGFR	CA	Mg	P	CRP	ESR	PCT	D-Dimer	LACTATE
1101229	1907	139	4.2		28	1.40	52.37	8.9			0.022				
1101231	0007	140	4.1		23	1.46	49.89	8.8							

DATE	TIME	GLUCOSE	HbA1C	CHOL	TG	HDL	LDL	KETONE	CK	CKMB	TRO-T	PT-P	APTT-P	NT-pro BNP
1101229	1907			166	92	45	101							
1101229	1908	164												

特殊檢查

病理報告

其它

出院時情況

- 3.改門診治療

出院指示

藥名	劑量	劑量單位	用法	頻次	數量	數量單位	現況	天數	單位
0.5mg Rivotril tab	1	TAB	PO	HS	8	TAB	TH	8	天
Amaryl tab 2mg	2	TAB	PO	BIDAC	32	TAB	TH	8	天
Duo Jardiance tab 12.5/850mg	1	TAB	PO	BIDAC	16	TAB	TH	8	天
Lipitor tab 20mg	1	TAB	PO	QD	8	TAB	TH	8	天
(口)Takepron OD tab 30mg	1	TAB	PO	QDAC	8	TAB	TH	8	天
DoxepinE cap 25mg	1	CAP	PO	HS	8	CAP	TH	8	天
OCAS Harnalidge 0.4 mg	1	TAB	PO	QDAC	8	TAB	TH	8	天
Nebilet tab 5mg	0.5	TAB	PO	QD	4	TAB	TH	8	天
Ultracet tab	1	TAB	PO	BIDPRN	16	TAB	TH	8	天
Actos tab 30mg	0.5	TAB	PO	QD	4	TAB	TH	8	天

出院計劃

- 門診日期
  - date on 2022/01/11

主治醫師 2062F 蔣鋒帆 / 住院醫師 2339C 蔡濬樟

正式摘要(電子簽章：000000233508987)

W83 / W83

doctorRank:B
